# The role of three-dimensional reconstruction in laparoscopic partial nephrectomy for complex renal tumors

**DOI:** 10.1186/s12957-019-1701-x

**Published:** 2019-09-11

**Authors:** Jipeng Wang, Youyi Lu, Gang Wu, Tianqi Wang, Yongqiang Wang, Hongwei Zhao, Zhongbao Zhou, Jitao Wu

**Affiliations:** Department of Urology, The Affiliated Yantai Yuhuangding Hospital of Qingdao University, NO. 20 East Yuhuangding Road, Yantai, 264000 Shandong China

**Keywords:** Laparoscopic partial nephrectomy, Three-dimensional reconstruction, Renal function

## Abstract

**Background:**

To evaluate the role of three-dimensional (3D) reconstruction technique in renal function protection and ipsilateral parenchymal mass preserved after laparoscopic partial nephrectomy (LPN) in patients with complex renal tumor (R.E.N.A.L.score ≥ 8).

**Methods:**

A retrospective study enrolling 49 patients who suffered from RCC and underwent LPN at our center, from October 1, 2017, to October 31, 2018. Twenty-one patients (group A) underwent LPN with the 3D reconstruction technique before surgery, and the other 28 patients (group B) not. Preoperative and postoperative ipsilateral parenchymal mass volume and ipsilateral glomerular filtration rate (GFR) were analyzed 3–5 days prior and 3 months after PN. In order to compare the two groups, Mann-Whitney *U* test and chi-square tests were performed. The main limitation of this technique is that the volume calculations are partly performed manually.

**Results:**

All patients’ median renal score was 10 with no difference between the two groups (*P* = 0.89), and the median tumor size of the two groups was 3.2 cm (group A) and 3.3 cm (group B) respectively (*P* = 0.14). In addition, the median warm ischemia time of the two groups was 21 min (group A) and 26 min (group B) (*P* = 0.003). In group A and group B, the rate of preserved global GFR was 88% and 86% (P = 0.06), preserved ipsilateral GFR was 80% and 77% (*P* = 0.01), and preserved ipsilateral parenchymal was 84% and 80% (*P* = 0.03) separately.

**Conclusion:**

3D reconstruction technique was a beneficial method for more renal function and more preserved renal parenchymal mass volume after LPN.

**Trial registration:**

Yantai Yuhuangding Hospital, YHD[2017]212. Registered 1 January 2017 (prospectively registered), http://www.ytyhdyy.com/nav/103.htm.

## Background

Partial nephrectomy (PN) was recommended for patients who suffered localized renal cell carcinoma (RCC), especially for cT1a tumors (according to the Union for International Cancer Control TNM staging system), on the account of preserving more nephron and improving the postoperative renal functional outcomes [1–5]. However, surgeons occasionally had to transform the PN into the radical nephrectomy (RN) for some complex renal tumors. Therefore, the renal functional outcomes became uncertain. Additionally, a recent research indicated that RN can improve the probability of suffering chronic kidney disease (CKD) and decrease the survival rate [6]. So, it is crucial to preserve more nephron for the RCC patients in the early stage.

Normally, two-dimensional (2D) computed tomography (CT) has occupied an important position in diagnosing the renal tumors [7]. However, traditional CT images were abstract and weak in describing anatomic structure for some complex tumors, such as renal hilar tumor and renal endophytic tumor. The actual position between tumor peripheral vessels was ambiguous, which increased the risk of additional damage in surgery. In recent years, one technology named three-dimensional (3D) CT reconstruction was applied to operation with the aim of decreasing the surgical accessory injury, maximizing the surgical accuracy, and protecting the postoperative organic function. In addition, this technique was comprehensively used in liver surgery [8], while seldom cases were reported in renal surgery.

In this study, we retrospectively analyzed renal functional outcomes after laparoscopic partial nephrectomy (LPN) as performed by an experienced surgeon, and compared preserved renal parenchymal volume after LPN by 3D reconstruction technique and traditional 2D CT scan separately.

## Methods

### Patients

The study protocol was approved by the Ethics Committee of the Affiliated Yantai Yuhuangding Hospital of Qingdao University (Yantai, Shandong), and informed consent was provided for the patients if they accepted the technique. From October 1, 2017, to October 31, 2018, 49 patients were required to take the abdominal CT scans with angiography and undergo LPN in our institution. Specifically for this research, since May 31, 2018, prior to the intervention, the eligible patients were told to take 3D reconstructive technique. So, the patients were divided into two groups (group A vs group B) in accordance with whether to take 3D reconstruction.

### Approach

In our study, we evaluated the complexity of tumors by R.E.N.A.L. score [9]. All surgeries were performed by an experienced urologist (accomplished the LPN more than 300 procedures); besides, the choice of intraperitoneal or retroperitoneal route was decided by the surgeon’s experience and patients’ characteristics. For each patient, we collected the basic indices, such as age, sex, body mass index (BMI), and Charlson comorbidity index (CCI); clinical indices, such as clinical tumor size, tumor growth pattern, and tumor’s R.E.N.A.L. score; perioperative indices, such as operative time, ischemia time, and estimated blood loss (EBL); and postoperative indices, such as pathologic tumor stage and positive margins. All kinds of parameters were supposed to reflect patients’ conditions adequately.

In addition, all enrolled patients were required to undergo examination for glomerular filtration rate (GFR) 3–5 days prior and 3 months after PN to evaluate the functional outcomes. The contrast-enhanced CT was supposed to be taken 3 months after PN to assess preserved parenchymal mass within the operated kidney. All operations of the two groups utilized the same kind of harmonic scalpel; besides, the dosage of the hemostatic agent was identical. Every case of our study utilized the “intraoperative doppler usg” during the operation, and the surgery only clamped the renal artery during the operation. Additionally, all of the volume (tumor and parenchymal mass) was calculated by cylindrical volume ratio method named Syngo Studio imaging software (Siemens, Washington, D.C.) [10], and the GFR was estimated by Modification-Diet-Renal-Disease-2 [11], and all patients were required to have nuclear renal scans (99mTc-mercaptoacetyltriglycine) to estimate the renal function within the operated kidney as the previous study described [12], because all 49 patients have two kidneys.

### Preparation for PN

**Fig. 1:**
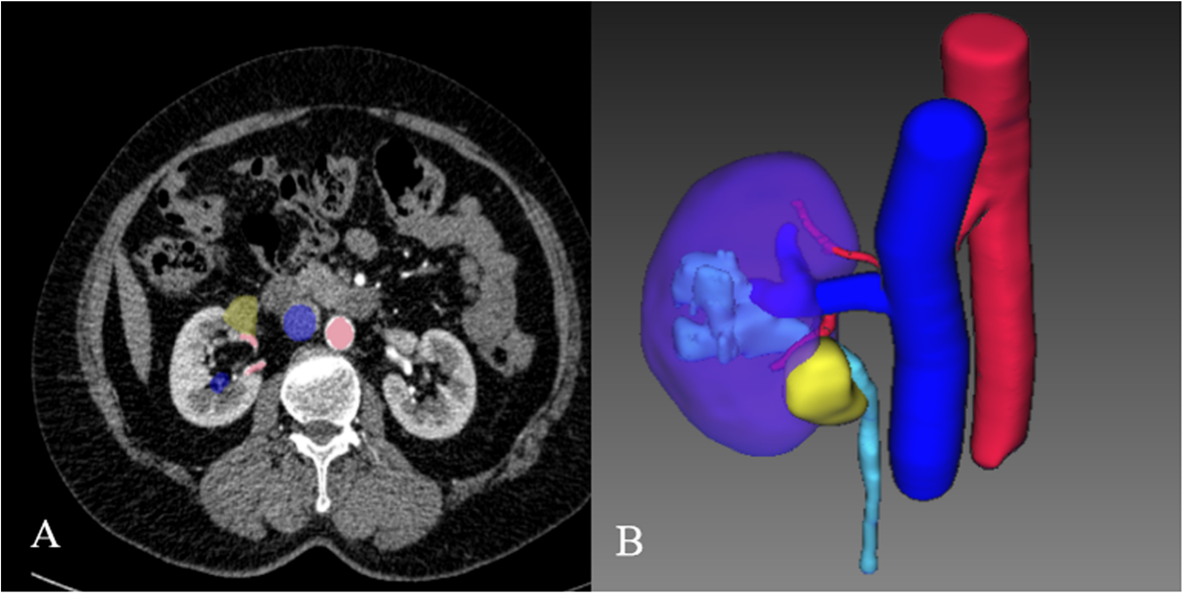
Renal hilar tumor’s 3D reconstruction and traditional CT scans. **a** General CT scans; red reflects artery, blue reflects vein, and yellow reflects tumor. **b** The reconstructive urinary system which include lesion, kidney, collective system, and blood vessels

In group A, tumors were described in coronal plane, vertical plane, and the cross-section by the 3D reconstructive technique (IQQA; EDDA Technology, Princeton, NJ, USA). Compared with the traditional CT scans, the tumors’ accurate location, peripheral vessels, and collective system were easily acquired [13, 14]. The profile of tumor was vividly presented to the surgeon (Fig. 1 describes the difference between traditional CT scans and 3D reconstructive CT scans). In addition, the reconstructive image was similar to the real anatomic structure. The surgeon watched the reconstructive CT on a normal display and analyzed the disease before the operation, which helped the surgeon accomplish a clear impression for complex lesion.

**Fig. 2:**
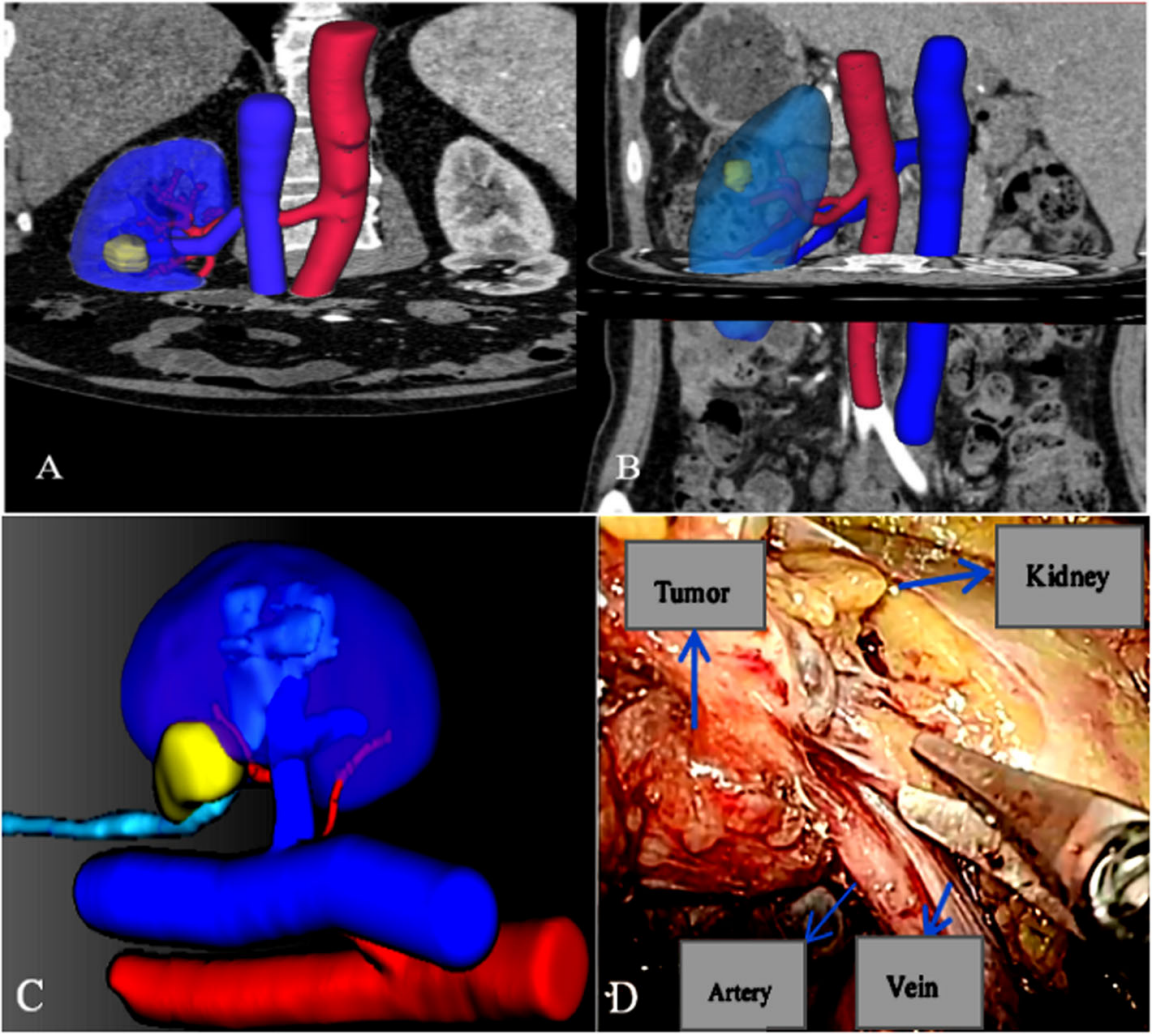
Two cases of completely endophytic renal tumors; the reconstructed model and real operative visual. **a** A completely endophytic tumor was located near the renal hilar. **b** A completely endophytic renal tumor was located in the upper pole. **c** Renal hilar tumor’s reconstruction, the tumor near a tiny renal artery. **d** Real operative visual showing the kidney, artery, and vein, and the relationship was alike with reconstructive image

When it came to traditional CT scans, the mass’s diameters can only be measured by ordinary 2D images, and single dimensionality image presented an abstract impression for the surgeon. For some complex renal tumors, especially the endophytic tumor, the exact comparative location between the mass and vessels can only be detected by surgeon’s experience, which occupied plenty of time for surgical processing, increasing the anesthesia risk. Figure 2 presented two kinds of completely endophytic renal tumor after being reconstructed. Additionally, tumors located in renal hilar may associate with some tiny, secluded vessels which were easily neglected and injured during operation. Hemorrhagic shock after surgery is likely to happen once surgeon neglected these small vessels injured.

### Statistical analysis

We utilized the Statistical Package for the Social Sciences (SPSS) version 25.0 (IBM, Armonk, NY, USA) to carry out the data analysis. All of the continuous variables (e.g., warm ischemia time, operative time, clinical tumor size, GFR) were described by median and inter-quartile range (IQR). The significance between categorical variables such as sex, tumor growing patterns, and pathologic tumor stage was analyzed by chi-square test. Besides, the significance of continuous arguments was compared by using the Mann-Whitney *U* test. Any differences between variable variate were significant if the *P*<0.05.

## Result

### Patient characteristics

All 49 patients accomplished undergoing the LPN with available data to study the global and ipsilateral GFR; operated renal parenchymal mass was preserved; besides, no patient died during the perioperative period. Group A included 21 patients who finally undertook the 3D reconstructive technique while other 28 patients were included in group B. In the two groups, patients’ median age was 56 and 60, respectively (*P* = 0.3), and the male’s retention occupied 71% and 61% (*P* = 0.4). All 49 patients’ median renal score was 10 (IQR 8–12 versus IQR 8–11, *P* = 0.89), and the median tumor size of patients in the two groups was 3.2 cm (IQR 2.1–4.3 cm) and 3.3 cm (IQR 2.3–4.2 cm), which showed no significance (*P* = 0.14). Additionally, renal hilar tumor and renal endophytic tumor were present in 15 and 6 patients in group A, while 15 and 13 patients in group B (*P* = 0.20), respectively, and the different distribution of renal hilar tumor between the two groups had no significance (*P* = 0.19), so as the endophytic tumor (*P* = 0.21). For operative time, 112 min (IQR 97–117 min) and 115 min (IQR 103–128 min) were respectively presented in group A and group B (*P* = 0.06). With regard to patients’ pathologic tumor stage, pT1a and pT1b were filed in 12 and 9 patients in group A while 21 and 7 patients in group B (*P* = 0.23). Patients who accepted this technique had significant decrease in estimated blood loss (129 mL, IQR 100–200 mL versus 160 mL, IQR 120–240 mL, *P* = 0.04). Compared with group B, patients had a shorter warm ischemia time in group A, and the median ischemia time was 21 min (IQR 16–25 min) and 26 min (IQR 21–28 min) respectively (*P* = 0.003). The tumor section time (9 min, IQR 6–13 min versus 13 min, IQR 13–16 min, *P* = 0.002) and renal suturing time (11 min, IQR 8–15 min versus 16 min, IQR 11–18 min, *P* = 0.001) also had an obvious decrease. Table 1 described the detailed patients’ characteristics data. Moreover, only one patient who suffered an endophytic tumor got a positive margin in group B.

**Table 1:** Patient characteristics

Variable	Group A	Group B	*P*
No. of patients	21	28	
Age (years), median (IQR)	56 (45–68)	60 (48–72)	0.3
Male, *n* (%)	15 (71%)	17 (61%)	0.4
BMI, kg/m, median (IQR)	23 (19–28)	24 (21–28)	0.4
CCI, median (IQR)	2 (1–3)	1 (0–2)	0.6
Clinical tumor size (cm), median (IQR)	3.2 (2.1–4.3)	3.3 (2.3–4.2)	0.14
R.E.N.A.L. score, median (IQR)	10 (8–12)	10 (8–11)	0.89
Tumor growth pattern (%)			0.20
Renal hilar tumor	15 (71)	15 (53)	0.19
Endophytic	6 (29)	13 (47)	0.21
Warm ischemia time (min), median (IQR)	21 (16–25)	26 (21–28)	0.003
Tumor resection time (min), median (IQR)	9 (6–13)	13 (10–16)	0.002
Suturing time (min), median (IQR)	11 (8–15)	16 (11–18)	0.001
EBL (mL), median (IQR)	129 (100–200)	160 (120–240)	0.04
Operative time (min), median (IQR)	112 (97–117)	115 (103–128)	0.06
Pathologic tumor stage (%)			0.23
pT1a	12 (73)	21 (65)	
PT1b	9 (27)	7 (35)	
Positive margins (%)	0 (0)	1 (2)	0.4

*BMI* body mass index; *CCI* Charlson comorbidity index; *IQR* interquartile range; *R.E.N.A.L.* (R)adius (tumor size as maximal diameter), (E)xophytic/endophytic properties of tumor, (N)earness of tumor deepest portion to collecting system or sinus, (A)nterior (a)/posterior (p)descriptor and (L)ocation relative to polar lines; *EBL* estimated blood loss

### Functional outcomes

For functional outcomes of the two groups, the median global preoperative GFR was 71 mL/min/1.73m^2^ and 73 mL/min/1.73m^2^ (*P* = 0.57) while the postoperative global GFR was 66 mL/min/1.73m^2^ and 64 mL/min/1.73m^2^ (*P* = 0.85). Meanwhile, no significance was found in preserved global GFR (the median preserved rate of GFR was 88% and 86% respectively, *P* = 0.06). The data of our letter inferred the organic compensation balanced out some functional regression for global functional outcomes. With regard to the operated kidney, the preoperative median ipsilateral GFR had no significance (44 mL/min/1.73m^2^ versus 42 mL/min/1.73m^2^, *P* = 0.70). However, the postoperative GFR of group A was higher than that of group B (36 mL/min/1.73m^2^ versus 33 mL/min/1.73m^2^, *P* = 0.04); besides, comparing with group B, patients in group A had a higher preserved GFR (80% versus 77 %, *P* = 0.01). For preoperative ipsilateral parenchymal mass, the median volume was present 168 cm^3^ and 171cm^3^ in group A and group B respectively (*P* = 0.61). Of notes, there was a significant difference in the postoperative ipsilateral parenchymal mass (median volume 157 cm^3^ versus 140 cm^3^, group A versus group B, *P* = 0.02). The patients in group A had a higher percentage of preserved kidney (84% versus 80%, group A versus group B, *P* = 0.03). The detailed functional outcomes were presented in Table 2.

**Table 2:** Renal function and parenchymal mass preserved

Variable	Group A	Group B	*P* (value)
No. of patients	21	28	
Analysis of global function (median, IQR)			
Preoperative global GFR, mL/min/1.73m^2^ (median, IQR)	71 (58–95)	73 (62–93)	0.57
Postoperative global GFR, mL/min/1.73m^2^ (median, IQR)	66 (49–83)	64 (47–81)	0.85
Global GFR preserved (%) (median, IQR)	88 (84–95)	86 (81–90)	0.06
Analysis focused on the operated kidney (median, IQR)			
Preoperative ipsilateral GFR, mL/min/1.73m^2^ (median, IQR)	44 (35–53)	42 (32–55)	0.70
Postoperative ipsilateral GFR, mL/min/1.73m^2^ (median, IQR)	36 (25–45)	33 (24–42)	0.04
Ipsilateral GFR preserved (%) (median, IQR)	80 (67–89)	77 (63–84)	0.01
Preoperative ipsilateral parenchymal mass, cm^3^ (median, IQR)	168 (147–200)	171 (159–198)	0.61
Postoperative ipsilateral parenchymal mass, cm^3^ (median, IQR)	157 (128–186)	140 (112–162)	0.02
Ipsilateral parenchymal mass preserved (%) (median, IQR)	84 (77–92)	80 (74–83)	0.03

*IQR* interquartile range, *GFR* glomerular filtration rate

## Discussion

PN was recognized as the gold standard for dealing with localized renal tumor [1], because it had a lower risk for CKD and higher overall survival rate compared to RN verified by several multi-center retrospective researches [6, 15, 16]. In addition, the quantities of preserved nephron were reported to be a fatal element which influenced postoperative renal functional outcomes if the extended warm ischemia could be avoided [17–20]; therefore, methods to protect the nephron became crucial. With respect to some complex renal tumors (R.E.N.A.L.score ≥ 8), especially entirely endophytic renal tumors and renal hilar tumors, additional normal parenchyma was always removed in order to get a complete surgical margin. Wu et al. did a retrospective analysis which indicated removing additional parenchymal mass increased the risk to suffer from CKD after PN and the non-renal cancer-related survival (NRCRS) would be reduced [6]. Besides, functional decline also occurred during parenchymal resection and reconstruction associated with tumor excision if lesions were surrounded by complex and secluded vessels. In this study, 3D reconstructive technique accurately reconstructed the anatomical structure and presented the tiny vessels and tissues around the tumor, which was proved to be a beneficial method to preserve more parenchymal mass and postoperative ipsilateral GFR.

According to the American Urological Association (AUA) guideline, patients were supposed to take the contrast-enhanced CT before surgery to notarize the location of the lesion. However, 2D CT scans were not the most sufficient preoperative preparation for some complex lesions. Cases were reported to suffer from severe complications in several kinds of medical center after PN (hemorrhagic shock or positive margin); all mentioned cases had the same characteristics that the tumor was almost endophytic or neared from renal artery branch. In addition, Porpiglia et al. had indicated high accuracy 3D reconstructive technique succeeded in selectively clamping the branches of renal artery during long-time robot-assisted partial nephrectomy (RAPN) for complex renal masses, maximally decreasing the renal damages caused by ischemia [14]. Moreover, 3D reconstructive technique was widely reported to be effectively used for preoperative evaluation and surgical planning in hepatic alveolar echinococcosis (HAE) [21]. Above all, 3D reconstructive technique had the huge potential to explore for PN.

In our research, 21 patients finally underwent the 3D reconstructive technique, both of the EBL and warm ischemia time had a significant decrease. The result of our study concluded that 3D reconstruction contributed to the tumor resection part and renal suturing part of the surgical procedure. Additionally, the median EBL of group A was only 129 mL with shorter warm ischemia time and more preserved ipsilateral parenchymal mass. Besides, none of the patients were reported to have positive margin in group A. When removing the tumor, a preciser location and volume helped the surgeon avoid damaging the normal tissue as little as possible, so that the renal parenchymal mass can be improved as much as possible. Additionally, some tiny peripheral vessels may be injured during the section of tumor removing or reconstructing the kidney, causing hemorrhage during operation and making the operative visual misty, which added the warm ischemia time. However, with the help of 3D reconstructive technique, the tiny vessels which are around the lesion or included in the tumor can be discovered before the operation, which helped the surgeon avoid injuring the tiny vessels when removing the tumor or reconstructing the kidney and shortened the warm ischemia time for hemostasis. Hence, both of the tumor resection part and renal suturing part had an obvious decrease. Besides, protecting more vessels during operation can reduce the devascularized parenchymal mass, which improved the preserved renal parenchymal mass [22]. Hence, our letter’s data provided a powerful proof to indicate 3D reconstructive technique helped surgeon integrally removed the tumor with a minimal margin and a great postoperative renal function.

Clinical findings supported that preserved renal parenchymal mass has been becoming the most meaningful factor to influence function outcomes after PN compared with the warm ischemia time [22]. GFR and parenchymal mass were recognized to be two advantageous factors to estimate functional outcomes [23–25]. Previous data indicated volume loss after PN was derived from the parenchymal resection and reconstruction associated with tumor excision [26–29]. What can we conclude from this study is that higher postoperative ipsilateral preserved GFR and more preserved ipsilateral parenchymal mass of group A bring a reasonable expectation that 3D reconstructive technique avoid damaging extra parenchyma and protect tiny vessels from being injured during reconstruction associated with tumor excision. Figure 2c and d described two kinds of images in describing two patterns of renal hilar tumors and compared the reconstructed image with the real operative visual.

Recently, research challenged that the ischemia injury was the primary determinant of ultimate function after PN; besides, Volpe et al. and Thompson et al. pointed that patients who underwent PN had a higher risk of suffering renal function decline if the warm ischemia time was at least more than 25 min [17, 30]. Data of our study presented the ischemia time was 21 min and 26 min, respectively, the shorter ischemia time of group A may not prove better functional outcomes after PN, but optimized the tumor excision and less surgical difficulty.

Limitations of this study included the nature of retrospective design and analysis, and our study was non-randomized. In addition, outcomes within one single medical center and the 49 patients in the study scale might produce degree of bias. The background differences cannot be controlled entirely, which might influence the functional outcomes, and a long-term and larger-scale study was supposed to be performed to analyze further functional outcomes of patients. The strength of our study is that all of the patients underwent LPN, and we can ignore the impact for surgical options, paying much attention to the postoperative functional outcomes. Moreover, this is the first contrastive analysis to focus on the preserved ipsilateral parenchymal mass and evaluate functional outcomes between 3D reconstructive and traditional CT scans after LPN.

## Conclusion

In conclusion, 3D reconstructive technique helps surgery get more comprehensive information before operation. In addition, more preserved renal parenchymal mass and shorter warm ischemia time contributed to achieve great renal function, which indicted that technique is worth to generalize in LPN. Moreover, more advanced technique is supposed to be applied for the operation and optimizing the functional outcomes after LPN; more lager sample size studies are required to validate our results.

## Data Availability

All data generated or analyzed during this study are included in this published article.

## Abbreviations

2D: Two-dimensional
3D: Three-dimensional
AUA: American Urological Association
BMI: Body mass index
CCI: Charlson comorbidity index
CKD: Chronic kidney disease
CT: Computed tomography
EBL: Estimated blood loss
GFR: Glomerular filtration rate
IQR: Interquartile range
LPN: Laparoscopic partial nephrectomy
NRCRS: Non-renal cancer-related survival
PN: Laparoscopic partial nephrectomy
R.E.N.A.L.: (R)adius (tumor size as maximal diameter), (E)xophytic/endophytic properties of tumor, (N)earness of tumor deepest portion to collecting system or sinus, (A)nterior (a)/posterior (p)descriptor and (L)ocation relative to polar lines
RAPN: Robot-assisted partial nephrectomy
RCC: Renal cell carcinoma
RN: Radical nephrectomy
SPSS: Statistical Package for the Social Sciences
